# Synergies between Fibrillated Nanocellulose and Hot-Pressing of Papers Obtained from High-Yield Pulp

**DOI:** 10.3390/nano13131931

**Published:** 2023-06-25

**Authors:** Carlos Negro, Gunilla Pettersson, Amanda Mattsson, Staffan Nyström, Jose Luis Sanchez-Salvador, Angeles Blanco, Per Engstrand

**Affiliations:** 1 Department of Chemical Engineering and Materials, University Complutense of Madrid, Avda Complutense s/n, 28040 Madrid, Spainablanco@ucm.es (A.B.); 2 Department of Engineering, Mathematics and Science Education (IMD), Mid Sweden University, SE-85170 Sundsvall, Swedenamanda.mattsson@miun.se (A.M.); per.engstrand@miun.se (P.E.)

**Keywords:** hot-pressing technology, microcellulose, cellulose nanofibers, nanocellulose, high-yield pulp, CTMP, paper quality, packaging

## Abstract

To extend the application of cost-effective high-yield pulps in packaging, strength and barrier properties are improved by advanced-strength additives or by hot-pressing. The aim of this study is to assess the synergic effects between the two approaches by using nanocellulose as a bulk additive, and by hot-pressing technology. Due to the synergic effect, dry strength increases by 118% while individual improvements are 31% by nanocellulose and 92% by hot-pressing. This effect is higher for mechanical fibrillated cellulose. After hot-pressing, all papers retain more than 22% of their dry strength. Hot-pressing greatly increases the paper’s ability to withstand compressive forces applied in short periods of time by 84%, with a further 30% increase due to the synergic effect of the fibrillated nanocellulose. Hot-pressing and the fibrillated cellulose greatly decrease air permeability (80% and 68%, respectively) for refining pretreated samples, due to the increased fiber flexibility, which increase up to 90% using the combined effect. The tear index increases with the addition of nanocellulose, but this effect is lost after hot-pressing. In general, fibrillation degree has a small effect which means that low- cost nanocellulose could be used in hot-pressed papers, providing products with a good strength and barrier capacity.

## 1. Introduction

High-yield pulps (HYP) have become a key material in sustainable products because of their resourcefulness and cost efficiency [1] and the potential to manufacture products which achieve certain important properties, such as a high bulk in paperboard and high light scattering in printing papers. They are produced at a yield of over 90% from different wood sources (or annual plants) by means of mechanical or combined chemical and mechanical processes [2,3]. One of the driving forces has been the fact that if high-yield processes can be used to a higher extent, it will be possible to manufacture more products from the same amount of wood since the corresponding yield for chemical pulps is around 50%. In addition, due to HYPs being a traditional and mature sector, industrial interest in this area is growing since new applications have been developed over the last few years. The market reduction, especially in printing products, due to digitalization, has forced the sector to search for other possible market strategies, transforming the processes and developing new HYP-based which includes their application for packaging to replace fossil-fuel-based plastic products [4]. Consequently, the research interest in improving the quality of papers obtained from HYPs has increased significantly in recent years [5,6].

The hot-pressing of various wood-based materials, such as wood, plywood, particle board, and fiberboard, has been used over the years to improve their material properties [7,8]. Song et al. (2018) showed that with a combination of chemical pre-treatment and hot-pressing, it is possible to densify the wood by almost 300% up to 1300 kg/m^3^ and to improve the tensile strength by over 1000% to 587 MPa [9]. This was obtained when 12% of the lignin was left in the wood prior to hot-pressing, which was optimal to maximize both density and strength values. On the other hand, Cristescu et al. (2015) concluded that temperature (up to 250 °C), compared to pressure and pressing time, is the most influential parameter in hot-pressing laminated beech, as it provides the highest density, strength, and lowest water sorption compared to the samples pressed at lower temperatures [10]. During hot-pressing, heat and mass-transfer processes interact with each other, in combination with deformation and chemical reactions, making the mechanism complex and challenging to investigate [11]. Therefore, simulations of hot-pressing have also been carried out to further understand the mechanisms at work [12,13].

Similar optimal amounts of lignin have been reported to maximize the reinforced effect of hot-pressed paper [14]. The benefit of using this hot-pressing method in combination with other methods to increase the strength of the sheet, such as the addition of chemicals, is that it is environmentally friendly and also that the sheet keeps its properties better over time [15]. In recent years, several studies have demonstrated that the hot-pressing of papers obtained with HYPs enhances their properties. Clear improvements are observed in both wet and dry tensile strength compared to non-pressed papers [16,17]. Joelsson et al. (2020) showed that the dry tensile strength of paper produced with various pulps, chemi-thermomechanical pulp (CTMP), thermomechanical pulp (TMP) and others, could be improved even by 100% when passing the paper through hot nips (200 °C, 6 MPa) [18]. Moreover, the wet strength increased dramatically from 2 kN·m/kg to about 16 kN·m/kg after hot-pressing. A high compression strength was also achieved, probably due to the high bending stiffness of the lignin-rich CTMP fibers compared to lignin-free chemical pulp fibers. Furthermore, promising results have also been found regarding other important properties of the paper for the final applications, such as water resistance. Contact angle measurements showed increased values for the hot-pressed paper samples, which suggests a more hydrophobic surface due to the increased density and smoothness of the paper [18,19,20].

The quality profile of these laboratory paper sheets is in line with or superior to which is demanded today for commercial advanced sustainable packaging paper materials (even without any addition of chemicals, such as wet-strength agents), where very high strength is highly prioritized, in products such as liners, and paper bags. Moreover, it was lately shown that it is possible to achieve wet-strength levels of over 50% of the dry-strength level by combining the hot-pressing of CTMP or lignin-rich kraft pulp with sizing agents such as ASA [15].

Since the different levels of improvement were related to the lignin content [21], the prerequisite to achieve these improved properties, both for wood and paper materials, is that optimal conditions during the press-drying of sheets are achieved, specifically that the temperature exceeds the softening temperature of lignin and hemicellulose. In 2021, Joelsson indicated that even better strength improvements might be possible at further increased press-drying temperatures [22].

On the other hand, in recent decades, it has also been proved that nanocellulose (NC) significantly increases mechanical and barrier paper properties, allowing the use of paper in applications covered in our days by other materials. Numerous studies related to the production, characterization, and use of NC in different applications of interest are described in the literature [23,24,25,26]. This is due to the excellent properties of this family of products, including the ability to form stable three-dimensional networks, its great mechanical resistance, colloidal properties [27], its high specific surface, adsorption capacity [28] or the capacity of functionalization, among others [29,30]. All these properties together with the great availability, and biodegradable, biocompatible and environmentally friendly properties of these materials make them of great interest in an endless number of applications like biomedicine [26,31], nanocomposites [32], reinforced inorganic matrix [33], rheology modifiers [34], aerogels and foams [35], food industry [36], energy [37], environmental [38], papermaking [39] and many others [40,41].

One of the most promising NC applications is in papermaking, especially in bulk and surface applications [39]. NC has been applied in papermaking due to its strong properties, mainly its high strength and mechanical properties; dry and wet strength; Z-strength and fracture toughness [42,43]; decreased porosity and permeability; enhanced barrier properties towards air, grease, water vapor and liquids; high oxygen-barrier performance [44]; its low density, enabling grammage reduction while increasing tensile strength [45]; increased filler retention and reduced surface roughness and water penetration [46]; decreased linting problems in printing papers [47]; and increased coloring efficiency of colored papers [48], water treatments [49], etc. Furthermore, as NC has a relatively high number of carboxyl groups, their reactivity make it possible to modify the hydrophilicity and charge surface to improve their potential applications in papermaking [39,50,51]. In addition, among all potential applications in papermaking, the use of NC as a reinforcement additive has been the most studied. NC-reinforced paper has the potential not only to improve conventional packaging paper, but also to progress to the substitution of non-biodegradable plastics obtained from fossil resources, as in the case of food packaging.

All published market studies about NC show that the market is expected to grow from USD 300 million currently to USD 800 million by 2026, which means an annual growth rate of more than 30% in the coming years, and this will certainly continue to grow in the future driven by the sustainability trend [52].

Therefore, the industrial and academic community are making a tremendous effort to unlock the potential for engineered sustainable materials based on NC, but several challenges remain related to the production, characterization and application of these products [53,54]. Factors such as process optimization and cost-effective production methods need to be further addressed. In this aspect, the in situ production and application of NC is considered. From this point of view, NC produced from fibers containing lignin, called lignocellulosic micro/nanofibers [55,56,57,58], and from recycled fibers, is attracting more attention since paper improvements were observed in z-strength, tensile index, tear index, burst index, E-modulus, strain at break, tensile stiffness, and air resistance [59,60].

The aim of this manuscript is to investigate the synergic effect of using fibrillated cellulose, containing micro and nanofibers (CMNFs), as a bulk additive, and hot-pressing to improve the properties of the paper. The cellulose source to produce CMNFs was the waste from a liner paper machine using 100% recovered paper. To our best knowledge, the use of NC to further enhance paper properties in conjunction to hot-pressing is a novel approach that has not been reported in the literature yet.

## 2. Materials and Methods

### 2.1. Materials

Waste liner from a paper machine using 100% recycled paper was used as cellulose raw material to produce CMNFs. The reagents used for the TEMPO-mediated oxidation pretreatment were 2,2,6,6-tetramethylpiperidin-1-yl)oxyl (TEMPO) reagent (98 wt.%) supplied by Sigma-Aldrich (St. Louis, MO, USA), as well as NaBr (>98.5 wt.%), NaOH (>98 wt.%) and NaClO (12% *w/v*, previously titrated to obtain the actual concentration) which were supplied by Panreac AppliChem (Barcelona, Spain). Other reagents used for the characterization of CMNFs were NaCl (>99.5 wt.%), NaOH (>98 wt.%), H_2_SO_4_ at 98 wt.% and crystal violet (>90.0 wt.% anhydrous basis) supplied by Merck (Madrid, Spain), and 0.1 wt.% Poly-L-Lysine solution with a molecular weight > 70,000 by Electron Microscopy Sciences (Hatfield, PA, USA).

For HYP production, a standard bleached flash dried spruce CTMP (CSF 420 mL) from SCA Östrand mill (Timrå, Sweden) was used to prepare the paper hand sheets.

### 2.2. Methods

#### 2.2.1. Cellulose Pretreatments

To produce the CMNFs, first, liner was left to soak for 24 h to favor fiber swelling before disintegration into a pulp disintegrator (PTI, Vorchdorf, Austria) at 30,000 revolutions. Two pretreatments were used to obtain the CMNFs. On the one hand, refining was used as the mechanical pretreatment using a PFI mill manufactured by Hamjem Maskin AS (Hamar, Norway). The pulp consistency was adjusted to 10 wt.% and the pulp was subjected to 20,000 revolutions [61,62]. On the other hand, a TEMPO-mediated oxidation (5T-Oxidation) chemical pretreatment was performed in a 5 L glass reactor at 1 wt.% cellulose consistency with 0.1 mmol TEMPO/g pulp and 1 mmol NaBr/g pulp [63,64]. Then, 5 mmol NaClO/g pulp was added to start the reaction which was conducted at room temperature and with stirring conditions at 200 rpm. During the process, pH was controlled to 10, adding a 2 M NaOH solution dropwise until pH remained constant [65,66].

#### 2.2.2. Cellulose Treatment

**Figure 1:**
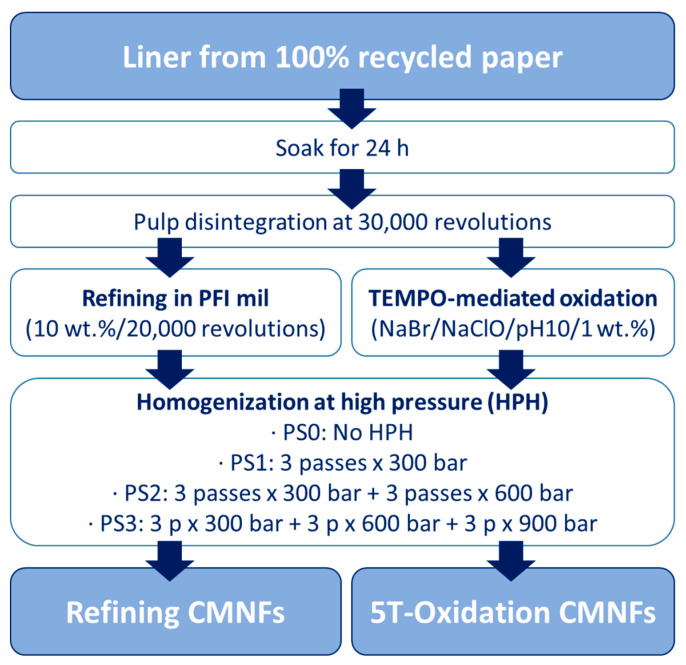
Scheme of CMNF production.

To produce CMNFs, pretreated pulps were mechanically fibrillated using a high-pressure laboratory homogenizer PANDA 2000 PLUS (GEA Niro Soavy, Parma, Italy) using pulp suspensions with a consistency around 1 wt.%. Figure 1 shows a scheme of the production of the CMNFs from the raw material. To achieve different nanofibrillation yields, four pressure sequences (PS) were carried out in the high-pressure homogenization (HPH). The PSs are described below from less intensive to more intensive:

- PS0: No homogenization of the fibers, only the pretreated samples.
- PS1: 3 passes of HPH at 300 bars.
- PS2: 3 passes of HPH at 300 bars and 3 additional passes at 600 bars.
- PS3: 3 passes of HPH at 300 bars, 3 passes at 600 bars and 3 passes at 900 bars.

#### 2.2.3. Chemical Characterization of the Raw Material Used to Produce CMNFs

First, the broke liner was dried to obtain the total solid content of the sample. Extractives, insoluble and soluble lignin, pectin, cellulose, hemicellulose, and ashes were measured by triplicate. Extractives of the sample were determined by Soxhlet extraction according to TAPPI T204 [67] whereas the ash content was determined by calcination at 525 °C, according to TAPPI T211 [68]. Soluble and insoluble lignin, pectin, cellulose and hemicellulose of raw materials were obtained following the NREL/TP-510-42618 standard [69]. A weight of 300 mg of the material was hydrolyzed with 3 mL of 72 wt.% H_2_SO_4_ for 1 h in a water bath at 30 °C. Then, 84 g of deionized water was added and introduced in an autoclave at 121 °C for 1 h. The hydrolyzed samples were vacuum-filtered. Insoluble lignin remained in the filter whereas the soluble lignin fraction was obtained by measuring the absorbance of the filtrate in the UV-Visible spectrophotometer at 240 nm. Hemicellulose, cellulose and pectin content were analyzed in the filtrate after neutralization with CaCO_3_ and passed through a 0.2 µm filter by using a modular HPLC device Jasco series 2000 (Jasco, Tokyo, Japan) [70].

#### 2.2.4. Characterization of CMNFs

The aspect ratio was obtained by the simplified gel point (*GP*) methodology based on the sedimentation of the fibers by using increments of the derivative at the origin of the curve C_o_ vs. *Hs*/*Ho*, as Equation (1) shows [71]. To determine the *GP* value, a 250 mL CMNF suspension was prepared using deionized water and 200 µL of crystal violet 0.1 wt.% to favor the sediment visualization [72]. The aspect ratio was calculated with Equation (2) according to Varanasi et al. (2013) [73], assuming a density of fibers around 1500 kg/m^3^ and the crowding number theory described by Martinez et al. (2001) [74]:

(1) GP=limHsHo→0⁡d∅odHsHo≈∅oi−∅o0HsHoi−HsHo0=∅oiHsHoi

(2) $$Aspect\;ratio=6.0\cdot \sqrt{\frac{1000}{GP\left(\frac{kg}{m^{3}}\right)}}$$

To characterize the morphology of CMNFs, optical microscopy (OM) and transmission electron microscopy (TEM) were used. Micro- and nanofibrils were visualized under 5× magnification using a Zeiss Axio Lab.A1 optical microscope and a color microscope camera Zeiss AxioCam eRc 5s (Carl Zeiss Microscopy GmbH, Göttingen, Germany). TEM analyses were carried out at the Centro Nacional de Microscopía Electrónica (Madrid, Spain) with a JEM 1400 microscope from JEOL (Tokyo, Japan). Samples were prepared adding 15 µL of 10% Poly-L-Lysine solution on a copper grid covered with a Formvar/carbon continuous layer. Then, 12 µL of 0.005 wt.% of CMNF suspensions were deposited and left to dry before TEM analysis [75]. To process the images, the program of public domain Image J was used to measure the diameter range of the different suspensions and evaluate the heterogeneity of the CMNFs. Transmittance readings of 0.1 wt.% diluted suspensions were measured in the wavelength of 600 nm on a UV–Vis Shimadzu spectrophotometer UV-160A using distilled water as reference. Finally, the carboxyl content of the suspensions was determined by conductometric titration according to Xu et al. (2022) [76].

#### 2.2.5. Hand Sheet Preparation and Testing

The CTMP pulp was soaked in hot water for around 1 h to soften the fibers (and favor fiber swelling). Then, they were hot disintegrated at 2% in solids using a PTI pulp disintegrator (Vorchdorf, Austria) at 30,000 revolutions according to ISO 5263-3 [77].

**Figure 2:**
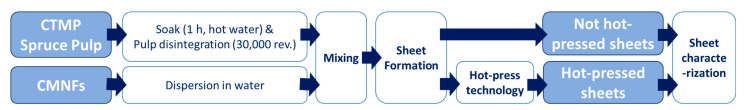
Scheme of hand sheet production.

Hand sheets of 100 g/m^2^ were prepared using a Rapid Köthen sheet former (Paper Testing Instruments, Pettenbach, Austria) according to ISO 5269-2 [78]. The sheets were dried in the drying plates in the Rapid Köthen until they reached a dry content of 65–70%, and thereafter put in a sealed plastic bag to maintain their moisture level. They were kept at 4 °C until hot-pressing. Figure 2 shows a scheme of the process of hand sheet production.

All sheets were characterized according to the different ISO standards after conditioning according to ISO 187 [79]. Density (kg/m^3^) was determined following ISO 534, grammage (g/m^2^) using ISO 536, and thickness (µm) with ISO 534 [80,81]. The determination of air permeability (mL/min) was measured according to the Bendtsen method following the ISO 5636 [82]. Tensile index (kN·m/kg) was conducted according to ISO 1924 and the wet tensile index (kN·m/kg) following the ISO 3781 [83,84]. Tear resistance (mN) was determined according to ISO 1974 and SCT (short-span compression test) (kN·m/kg) following ISO 9895 [85,86]. Error bars were calculated using the standard deviation between samples with the same conditions.

Paper samples were analyzed using a high-resolution SEM (Tescan Maya3-2016, TESCAN Brno, s.r.o., Brno, Czechia). All samples were prepared by sputtering them with a 5 nm thin layer of iridium prior to imaging. The applied electron beam voltage was 3.00 kV, and the beam intensity was 1.00. To obtain images of the structures at different scales, magnifications 200×, 1000×, and 2000× were used. The working distance to the sample was set to approximately 8 mm.

#### 2.2.6. Hot-Pressing Technology

**Figure 3:**
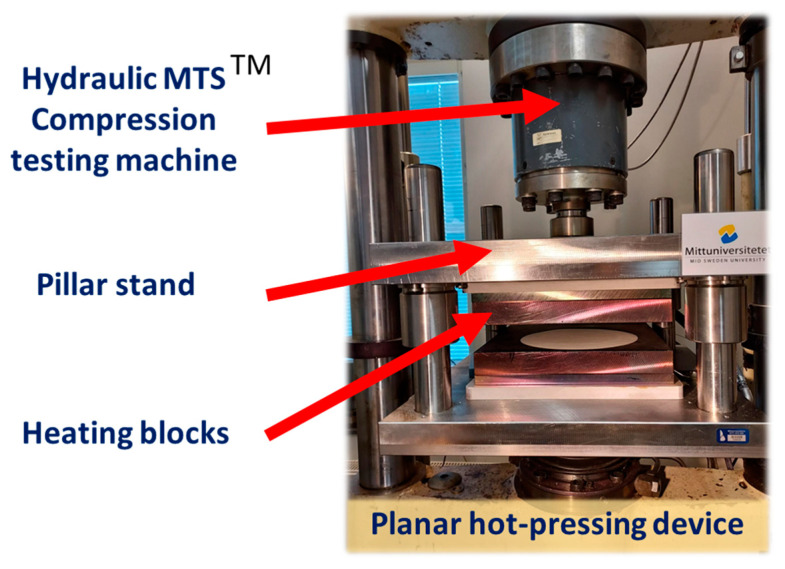
Planar hot-pressing unit with temperature and load controlled pressing plates built into an MTS equipment (developed) at Mid Sweden University.

The paper sheets were hot-pressed in a planar pressing equipment built at Mid Sweden University. This equipment mainly contains three parts: the heating blocks, pillar stand, and compression testing machine (see Figure 3). The heating blocks have a dimension of 300 × 300 × 40 mm and have three pockets in which flat electrical heating elements, each of 500 W, are inserted. Due to the thickness of 30 mm steel between elements and the pressing surface, variations in the temperature distribution will be at a minimum. The heating blocks are, via 20 mm thermal isolating plates, mounted in pillar rack ball brushings to ensure the best alignment between the blocks. In turn, the upper pillar rack is fixed to a hydraulic MTS^TM^ material testing machine loadcell and the lower part to the movable hydraulic piston rod. For the control of block temperature, each has a built-in thermocouple sensor connected to Eurotherm PID-type regulators which are limited to 300 °C. For the control of compression loads, MTS^TM^ RPC software is used for creating block-programmed load vs. time sequences up to 100 kN.

In these experiments, the pressing time was set to 3 s, and the pressure to 3.5 mPa. All experiments were carried out at 260 °C with a dry content of 65–70% on the sheets. The sheets were manually handled during the hot-pressing trials.

## 3. Results and Discussion

### 3.1. Characterization of the Cellulosic Raw Material and the CMNF Suspensions

Chemical characterization of the spruce CTMP used as the HYP and the broke liner used to produce the CMNFs is shown in Table 1, together with the composition of the oxidized pulp after 5T-Oxidation. Pulp after refining was not characterized due to the pretreatment only being mechanical. The raw materials (spruce and liner) were dried to obtain a total solid content amounting to 96.6 ± 0.2% and 96.8 ± 0.2% for liner and spruce, respectively. The ash content due to the presence of fillers in the recycled paper was elevated with an average value of 14.2%, whereas in the spruce this value was under 1% [87]. On the other hand, the content of total lignin in both raw materials was also elevated with an average value of 17.2% and 29.9%, indicating that only part of the initial lignin was removed to facilitate the separation of the virgin fibers, as usually performed in packaging paper. The cellulose content was similar in both cases with a 55.2 ± 0.9% in the liner and 48.8 ± 1.2% for the spruce CTMP. The hemicellulose content, formed in the chemical composition by arabinose and the overlapping of xylose, mannose, and galactose, was 10.6% in liner while the spruce almost doubled this value with 18.5%. Finally, the extractive content was under 2% for the liner and around 0.5% for the spruce whereas the pectin content was also low, around 1.5% in both cases. This last parameter was calculated as the galacturonic acid content in the hydrolyzed sample, but also the rhamnose, which majoritively proceeds from pectin, although to a lesser extent from hemicellulose. However, the content of rhamnose is insignificant in the sample under 0.4%, assuming all rhamnose came from the pectin [88].

The suspensions obtained after the different pretreatments and the mechanical homogenization at several intensities were characterized. The samples pretreated by refining have an proportion of solids of around 1–1.1% in the suspensions and the carboxyl group content was 0.46 ± 0.03 mmol/g pulp. In the case of pulps pretreated by TEMPO-mediated oxidation, the solid content was 1.1–1.2% and the carboxyl group content increased up to 1.16 ± 0.04 mmol/g pulp which suggested good fiber oxidation, with a remarkable increase similar to other studies [63,65,76]. The pulp had a higher content of ashes due to the production of salts as a parallel reaction during the oxidation [89]. In addition, the action of the oxidant removes part of the insoluble lignin and dissolves the cellulose at a higher proportion than the hemicellulose [64,90]. On the other hand, in neither of the pretreatments was a variation in the carboxyl groups observed with the intensity of the homogenization.

Table 2 shows the transmittance and aspect ratio of the obtained CMNF suspensions. Transmittance gives an idea of the homogeneity of the CMNFs fibers. When a sample has been highly fibrillated, the minimum possible diameter is reached, and the suspension becomes optically transparent at a test concentration (0.1 wt.%), with a transmittance near to 100% [91,92]. Aspect ratio refers to the relationship between the length and the width of the fibers. It is a fundamental parameter used to describe the morphology and physical properties of CMNFs, providing valuable information that influence their mechanical, thermal, and optical properties [72,93]. Samples obtained by a refining pretreatment have a low transmittance associated with a low light pass and the presence of more microfibrils than nanofibrils. In the case of chemically pretreated samples, the proportion of nanofibrils is higher and the value increases with the intensity of homogenization. It is interesting to point out that the pretreated sample with 5T-oxidation without homogenization (PS0), shows too high a transmittance value due to the sedimentation of the fibers, since the cellulose has not been subjected to any type of defibrillation. As expected, results of the aspect ratio show an increase with the intensity of homogenization. Comparing both pretreatments, it is observed that in the case of refining, the fibers are separated from the primary structure producing more ramified fibers which increase the aspect ratio values. In the case of 5T-Oxidation, besides cellulose oxidation, the oxidant also produces the breakage of the cellulose chains into smaller units as reflected in lower aspect ratios [72].

**Figure 4:**
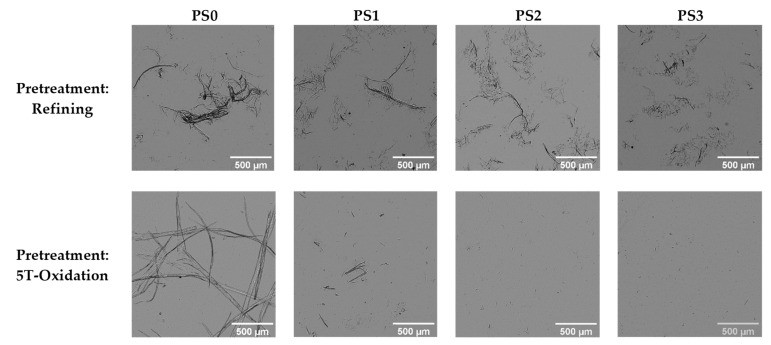
Optical micrographs (OM) at 5× magnification of the CMNF suspensions.

**Figure 5:**
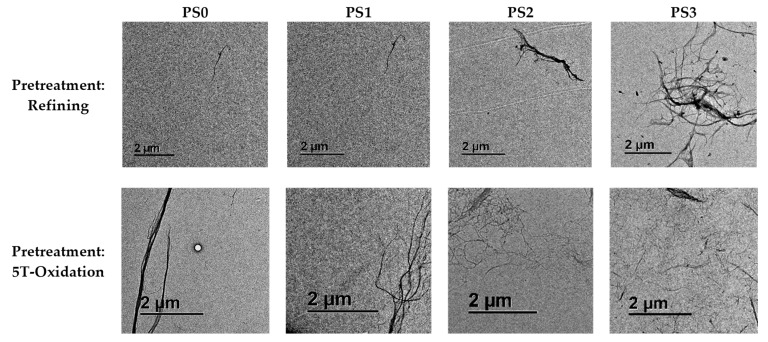
TEM micrographs at 1000× magnification of the CMNF suspensions.

Micrographs of the suspensions have been carried out using OM (Figure 4) and TEM (Figure 5) at 5× and 1000× magnification, respectively. At least five images of each microscopic technique were taken. In addition, the variety of magnifications allows us to also evaluate the degree of homogeneity of the fiber size. The microfibrils after the refining pretreatment (PS0) showed a diameter range from 3 to 40 μm as observed in the OM micrographs. However, TEM images show some thinner fibers of 100 nm but in a very small proportion. Only after six homogenization passes (PS2) was is possible to observe a representative number of nanofibers in the samples. After the most intensive mechanical treatment (PS3), the diameter range of the sample was reduced to a range between 20 nm and 6 μm. In the case of the fibers treated with TEMPO-mediated oxidation, the diameter range without homogenization was more heterogeneous with micro and nanofibrils from 30 nm to 60 μm. Only after the sample was subjected to the mechanical treatment (PS1) did cellulose start to fibrillate in a notable way, being mostly nanofibers. After nine homogenization passes these CMNFs were more homogeneous with a diameter range between 15 and 70 nm.

### 3.2. Paper Properties

#### 3.2.1. Air Permeability

The main property affected by the presence of NC was the reduction in air permeability, providing products with a good barrier capacity that is beneficial for applications in the packaging industry, especially for food packaging [94].

**Figure 6:**
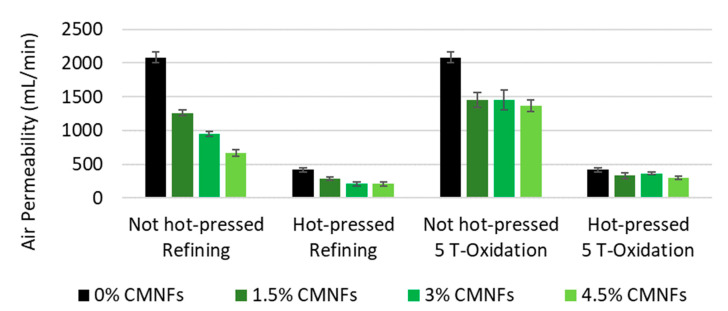
Influence of CMNF dosages on air permeability.

As it can be seen in Figure 6, in which CMNFs produced with PS2 homogenization sequence are used, the increase in fibrillated cellulose dosage significantly decreases air permeability by up to 68%, from 2.084 ± 75 mL/min to 670 ± 52 mL/min, with the addition of 4.5% of refined CMNFs in non-hot-pressed papers (NHP). The synergy effect on air permeability further increases after hot-pressing (HP) up to 50%, from 418 ± 32 mL/min to 211 ± 30 mL/min. This can be explained because at high temperatures, the fiber structure becomes more flexible and easier to compress, reaching a high sheet density, and this effect is accentuated with the presence of CMNFs. NC also acts by blocking the voids in the 3D network, reducing the ease of air-volume passage within, since compacted NC provides longer tracks for air molecules to pass through [95]. Air-permeability reduction is also related to a decrease in porosity with NC addition [96].

It is remarkable that it is possible to achieve a significant air permeability reduction at very-low-refining NC dosages (1.5%), decreasing by 40% and 32% in the NHP and HP papers, respectively. This suggests that a low NC dosage is enough to achieve the desired properties. It was also observed that the best results were obtained using a refining process during the pretreatment to produce CMNFs instead of a tempo-mediated oxidation process (TMO); this can be explained by the fact that with the refining process more flexible fibers with a higher fine content were obtained, leading to an easier network structure to compress.

**Figure 7:**
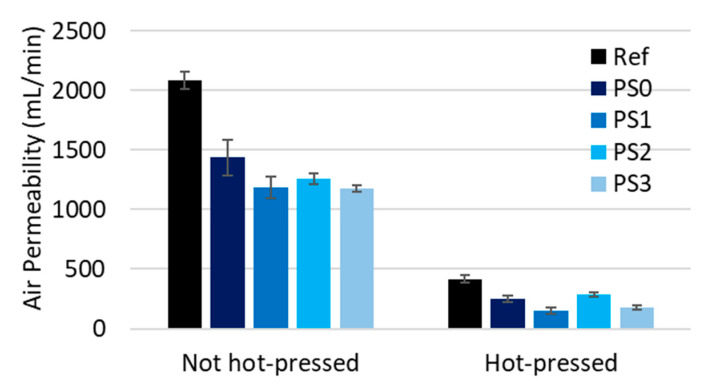
Influence of CMNFs on air permeability at dosage 1.5%.

At this low NC dosage (1.5%), a series of tests were performed to calculate the influence of the fibrillation degree on the reduction in air permeability using the fibrillated cellulose obtained using the refining pretreatment. Figure 7 shows that air permeability slightly decreases when the fibrillation degree increases but the improvement does not justify the use of highly fibrillated NC such as TMO. This is important because low-cost fibrillated NC could be used in hot-pressed papers. On the other hand, by increasing refining, it is possible to obtain denser products; this is due to the improvement in fiber flexibility and the higher content of fines.

The high differences between the hot-pressed and non-hot-pressed samples could be explained because of the collapse of the fiber network causing a higher density. In fact, the density of the hot-pressed papers reaches values around 800 kg/m^3^, almost double the average density of the non-hot-pressed papers 450 kg/m^3^. However, this is not the only factor as no differences were obtained in the densities of the non-hot-pressed sheet independently of the NC dosage or of the fibrillation degree. The reduction of bulk with NC addition can be also associated with intrinsic NC properties, the better fiber network accommodation entails more compacted papers [97].

The synergy between the use of fibrillated cellulose as a bulk additive and hot-pressing is very significant. Hot-pressing and fibrillated cellulose highly decrease the air permeability (80% and up to 68%, respectively) for refining the pretreated samples, due to the increased fiber flexibility, which increase up to 90% using the combined effect.

**Figure 8:**
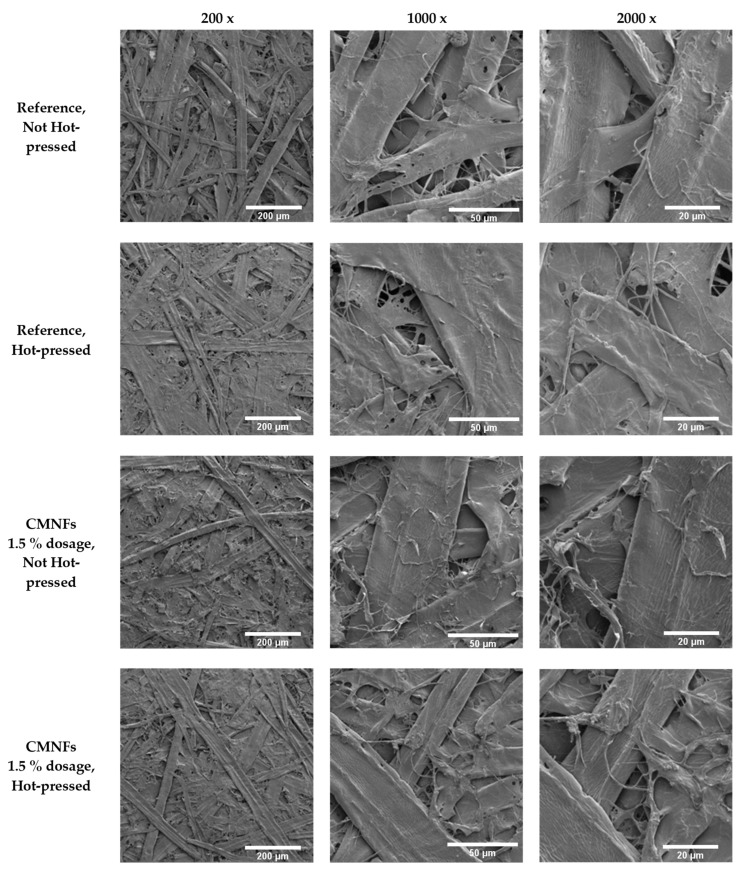
SEM images of different papers.

Figure 8 shows SEM images of different samples; as it can be observed, the surface of the samples containing CMNFs is covered by the fibrillated cellulose reducing and closing the voids formed in the 3D fiber network by the larger fibers and, as consequence, these papers have a lower air permeability.

#### 3.2.2. Dry Strength

**Figure 9:**
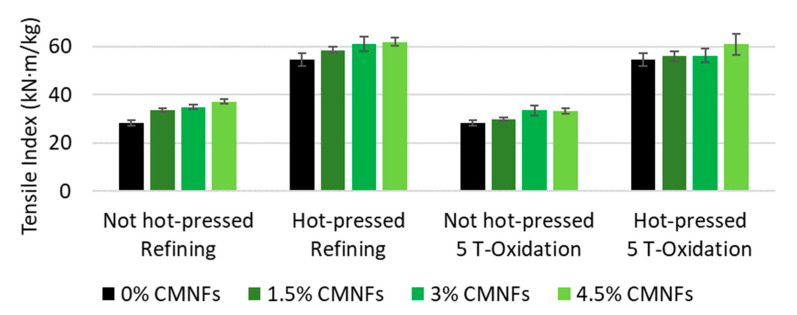
Influence of fibrillated nanocellulose on tensile index.

Figure 9 shows the increase in tensile index (tensile strength divided by the grammage of the sheet) when increasing the dosage of fibrillated cellulose produced with a PS2 homogenization sequence. The fiber network strength increased by increasing the number of bonds. The tensile index increased from 28.3 ± 1.2 kN·m/kg to 37.2 ± 1.0 kN·m/kg (31.4%) in non-hot-pressed papers and from 54.6 ± 2.7 kN·m/kg to 61.9 ± 1.8 kN·m/kg (13.4%) in hot-pressed papers when adding refining CMNFs. Due to their high aspect ratio and large relative surface area, CMNFs can enhance the bonding between fibers and even form a more crosslinked network [98].

The synergy between the use of fibrillated cellulose as a bulk additive and hot-pressing is very significant. In the case of dry strength, improvements up to 118% are obtained while individual improvements are up to 31% by CMNFs and 92% by hot-pressing (see Figure 9).

**Figure 10:**
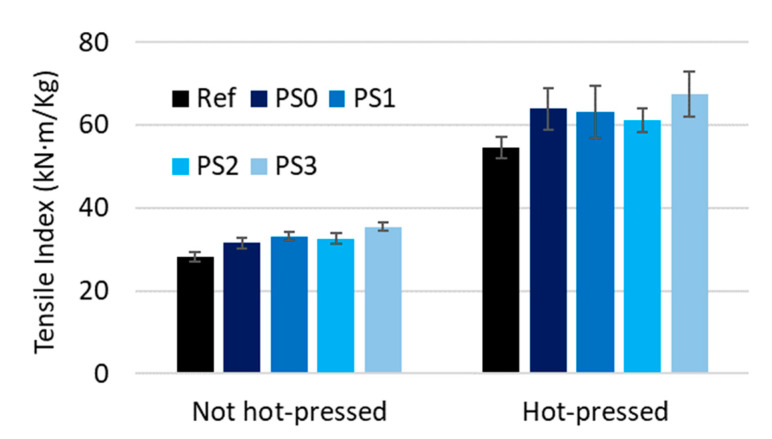
Influence of 1.5% of fibrillated cellulose on tensile index at different fibrillation degrees.

**Figure 11:**
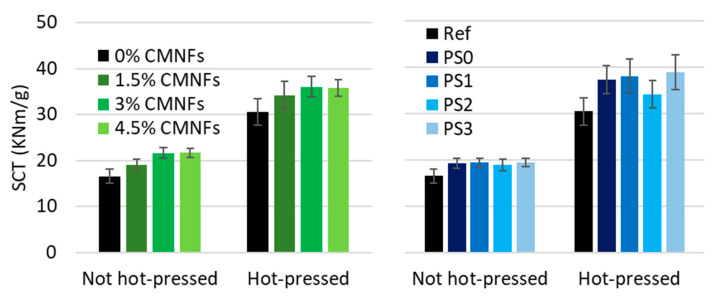
Influence of CMNFs on SCT.

It should be noted that the addition of fibrillated cellulose also increases the tensile index after hot-pressing but to a lower level. However, this increment is small in comparison to the effect of the hot-press itself, which almost doubles the tensile index. This effect is associated in part to the presence of lignin in the HYP [18]. Therefore, the fiber bonds are not the only responsible aspect of this increase after hot-pressing, supporting the theory that paper is strengthened by new covalent bonds, possibly in a crosslinking structure within the lignin and/or between the lignin and carbohydrates. This can also be demonstrated when applying 1.5% of refining CMNFs at different fibrillation degrees as it is shown in Figure 10. As expected, compression strength (SCT) followed the same pattern as tensile index as shows in Figure 11.

#### 3.2.3. Wet Strength

It is well known that independently of the type of pulp used to produce the paper, the inter-fiber bonding is unstable in water in untreated papers. As fibrillated cellulose has a high capacity to absorb water, normally the wet strength of paper containing NC decreases except in the cases in which the fibrillated material is treated to become hydrophobic.

**Figure 12:**
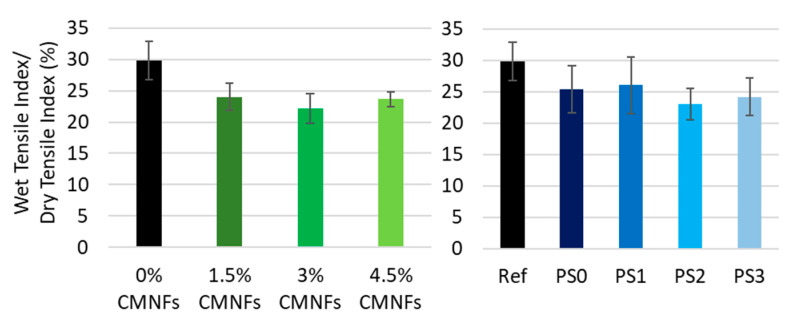
Influence of fibrillated cellulose on wet strength after hot-pressing technology.

As expected, results show that without hot-pressing, papers do not have wet strength. However, after application of hot-pressing technology, all papers, independently of the dosage and type of fibrillated cellulose, retain more than 20% of their wet tensile strength (See Figure 12). A paper is considered to have good wet strength properties when it retains more than the 15% of its dry tensile strength after being immersed in water [99]. The lower water uptake can be explained by a combination of factors:

1. The presence of fibrillated cellulose reduces the voids between fibers in the sheet becoming smaller or closed, providing more fiber-network bonding capacity [100]. The lignin content of CTMP could act acting to protect these fiber-fiber bonds at a high temperature.
2. Due to a more hydrophobic surface character of the paper after hot-pressing technology [101].
3. New water-resistant bonds such as covalent bonds are formed during hot-pressing [102].

Furthermore, CMNFs can retain more water using hydrogen bonding and within the 3D network than pulp fibers due to their large specific area and the high number of carboxyl groups they contain. Consequently, its addition can improve the hydrogen bonds between the cellulose fibers during the pressing process, thus increasing the web strength.

#### 3.2.4. Tear Resistance

**Figure 13:**
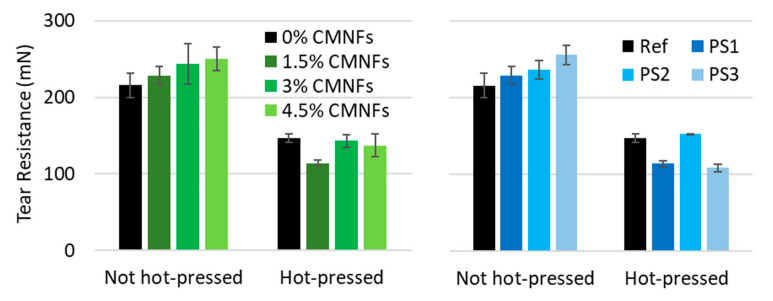
Influence of CMNFs on tear resistance.

One of the drawbacks of hot-pressing technology is that the tear index decreases because the paper obtained has a higher density and it is more rigid. It was decided to test if the addition of fibrillation material could avoid at least partially this loss in tear index. As can be seen in Figure 13, tear index increases with the addition of CMNFs maintaining the fibrillation degree in PS2 and further increases with the fibrillation degree maintaining the CMNF dose at 1.5% (Figure 13). Further improvements were achieved than previous studies by Guan et al. (2019) and Balea et al. (2019) demonstrated, which reported that NC content did cause a slight increase in the tear index [59,103]; however, this effect was lost after hot-pressing as the material was still very rigid, limiting the application of hot-press papers to those applications in which tear index values are not critical.

## 4. Conclusions

The synergy between the use of fibrillated cellulose as a bulk additive and hot-pressing has been assessed. This synergy effect is very significant, further improving the properties of hot-pressed papers. Hot-pressing and fibrillated cellulose highly decrease air permeability (by 80% and 68%, respectively) for refining pretreated samples, due to the increased fiber flexibility, which increases up to 90% due to the combined effects. In the case of dry strength, improvements up to 118% can be obtained while individual improvements were 31% by CMNF and 92% by hot-pressing. This effect is more pronounced for mechanical fibrillated cellulose. It is important to note that after hot-pressing, all papers retain a wet strength 22% higher than that of the dry strength, even at high NC dosages. Furthermore, the combined effect also increases the SCT values, related to paper’s ability to withstand compressive forces applied in a short period of time, by 110% while hot-pressing produces an increase of 84% compared to the reference paper. The tear index increases with the addition of nanocellulose, but this effect is lost after hot-pressing.

In general, the fibrillation degree has a low effect, which means that low-cost nanocellulose could be used in hot-pressed papers to provide products with a good strength and barrier capacity. Data show that it is possible to extend the application of cost-effective high-yield-pulp packaging through improving strength and barrier properties by using CMNF as a bulk additive and by hot-pressing the obtained papers. This shows that in hot-pressing technology, fiber bonding plays a key role, as well as the new covalent bonds formed at high temperature, possibly in a crosslinking structure within the lignin and/or between the lignin and carbohydrates. As lignin could play an important role in the enhanced properties of hot-pressed paper, it is recommended to explore the possibility of using lignin-based nanoparticles to obtain more knowledge on this theory.

## Figures and Tables

**Table 1:** Chemical composition of spruce CTMP as HYP and recycled liner for CMNF production before and after 5T-oxidation.

Chemical Composition (Dry Basis)	Spruce CTMP	Liner	Liner after 5T-Oxidation
Ash (%)	0.7 ± 0.2	14.2 ± 0.1	16.0 ± 0.5
Cellulose (%)	48.8 ± 1.2	55.2 ± 0.9	52.7 ± 1.5
Hemicellulose (%)	18.5 ± 0.9	10.6 ± 0.6	13.1 ± 0.4
Acid insoluble lignin (%)	25.6 ± 0.4	11.7 ± 0.4	7.1 ± 0.5
Acid soluble lignin (%)	4.3 ± 0.1	5.5 ± 0.1	9.0 ± 1.5
Pectin (%)	1.6 ± 0.3	1.2 ± 0.3	<0.5
Extractives (%)	0.5 ± 0.1	1.7 ± 0.3	1.9 ± 0.2

**Table 2:** Transmittance and aspect ratio of CMNF suspensions.

Homogenization Intensities	Transmittance		Aspect Ratio	
	Refining	5T-Oxidation	Refining	5T-Oxidation
PS0	4.5 ± 0.3	39.1 ± 2.2	86 ± 5	43 ± 2
PS1	4.6 ± 0.4	22.9 ± 0.1	127 ± 5	76 ± 3
PS2	5.5 ± 0.3	27.6 ± 0.6	127 ± 6	101 ± 7
PS3	9.5 ± 0.5	40.7 ± 0.2	128 ± 5	103 ± 8

## Data Availability

Data available on request.
